# ﻿The collection of the genus *Epepeotes* Pascoe, 1866 housed in the Natural History Museum, London (Coleoptera, Cerambycidae)

**DOI:** 10.3897/zookeys.1184.111728

**Published:** 2023-11-10

**Authors:** Guanglin Xie, Maxwell V. L. Barclay, Wenkai Wang

**Affiliations:** 1 Institute of Entomology, College of Agriculture, Yangtze University, Jingzhou, Hubei, 434025, China Yangtze University Jingzhou China; 2 Department of Life Sciences, Natural History Museum, London, SW7 5BD, UK Natural History Museum London United Kingdom

**Keywords:** Longhorned beetles, new distribution, new status, NHMUK, type material, zoological collections

## Abstract

Data on the collection of the genus *Epepeotes* Pascoe, 1866 deposited in the Natural History Museum, London are presented. A total of 23 species/subspecies, including type specimens of 18 names, of them 13 valid, are recorded. *Epepeotesuncinatuslineatopunctatus* Breuning, 1960 is restored to subspecies-level status. Lectotypes are designated for *Epepeotesuncinatusuncinatus* Gahan, 1888 and *Epepeotesandamanicus* Gahan, 1893. *Epepeotesluscusluscus* (Fabricius, 1787) is newly recorded in Cambodia and Singapore, and *Epepeotesuncinatusuncinatus* Gahan, 1888 is newly recorded in Bangladesh. Images of the type and other significant specimens are provided for 23 taxa, mainly for the first time.

## ﻿Introduction

The genus *Epepeotes* was established for the species *Lamialusca* Fabricius, 1787 by Pascoe (1866a). It currently consists of 39 species/subspecies widespread in Asia (Tavakilian and Chevillotte 2023). The following genera are considered as synonyms: *Diochares* Pascoe, 1866 [type-species: *Cerambyxfimbriatus* Olivier, 1795 (= *Cerambyxdesertusdesertus* Linnaeus, 1758)], *Mengelotes* Dillon & Dillon, 1941 [type-species: *Mengelotesambiguus* Dillon & Dillon, 1941 (= *Macrochenusambigenus* Chevrolat, 1841)] and *Falsomonohammus* Pic, 1943 [type-species: *Falsomonohammusdiverseglabratus* Pic, 1943].

This study is based on the collection of the Natural History Museum, London [formerly British Museum, Natural History (BMNH), and hereafter NHM or NHMUK], which houses 620 categorized specimens (not counting those not sorted) of 23 valid species/subspecies, of which 13 valid taxa and five junior synonyms are represented by type material. A record of all species is given below.

## ﻿Material and methods

The material examined in this study, already identified by earlier specialists, is deposited in the NHM. Verbatim quotation is used here for all labels of studied type specimens and the label text is given in single quotation marks. Labels of non-type specimens are summarized briefly, to provide the reader with information on the country of origin, locality, but more detail, such as acquisition numbers, collector names and month and year of collecting and other information, is generally not reproduced. Individual labels are separated by a semicolon, and data on different rows by a single slash. Additional and explanatory comments by the authors are given in square brackets. Abbreviations are used in the text for label text: h for handwritten, p for printed. In the list, species records are listed in chronological order of publication, different subspecies of the same species are presented together in chronological order.

Photographs were taken using a Canon 7D Mark II SLR camera with a Canon EFS 100 mm lens and edited using Adobe Photoshop 2020 release. Extended depth of field at magnifications was achieved by combining multiple images from a range of focal planes using Combine ZM or Helicon Focus software.

## ﻿Results

Data on the genus *Epepeotes* from the collection of the NHM are summarized on the basis of 620 observed specimens. The collection comprises 23 valid taxa, including types of 18 names, of them 13 valid and five invalid. Labeling details of each type specimen are reproduced verbatim without corrections or additions. Some additional information is provided as comments. The list of *Epepeotes* deposited in the NHM collection is given as follows.

### ﻿ Epepeotes desertus desertus

Taxon classificationAnimaliaColeopteraCerambycidae

(Linnaeus, 1758)

D15EEE0A-F964-52EA-8DE5-4001FCED40CB

Fig. 1a–c

Cerambyx desertus Linnaeus, 1758: 391. Type locality: America [wrong locality record].
Cerambix (Lamia) fimbriatus Olivier, 1795: 71 [misspelling].
Lamia lineator Fabricius, 1801: 283. Type locality: India.
Diochares fimbriatus : Pascoe 1866: 303.
Epepeotes desertus : Breuning 1943: 229.

#### Non-type material.

8 specimens. **India (5)**: E. India (1 male, 1 female), Goram (1 male, 2 females). **Indonesia (2)**: Ceram (1 female); Moluccas (1 male). **Country unknown (1)**: Pascoe collection (1): Mano (1 female).

**Figure 1.**
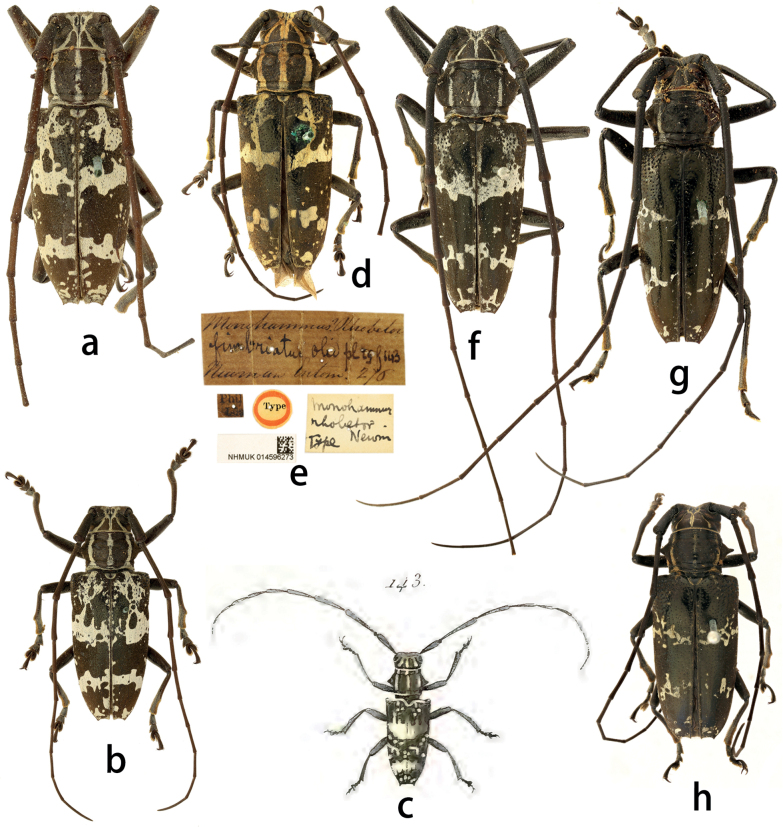
Habitus of *Epepeotesdesertus* (Linnaeus, 1758) **a–c***Epepeotesdesertusdesertus* (Linnaeus, 1758) **d–f***Epepeotesdesertusrhobetor* (Newman, 1842) **g, h***Epepeotesdesertusobscurus* (Aurivillius, 1926) **d, e** holotype **a, f, g** male **b, d, h** female.

### ﻿ Epepeotes desertus rhobetor

Taxon classificationAnimaliaColeopteraCerambycidae

(Newman, 1842)

CF29D9B8-7FD6-56E7-9184-192411FE5AB1

Fig. 1d–f

Monohammus ? Rhobetor [sic] Newman, 1842: 276. Type locality: Manila [Philippines].
Diochares desertus var. rhobetor : Aurivillius 1922: 81.
Epepeotes desertus s.- sp. rhobetor : Breuning 1943: 229.
Epepeotes desertus ssp. rhobetor : Breuning 1961: 326.
Epepeotes desertus rhobetor : Barševskis 2020: 180.

#### Type material.

**Phllippines. *Holotype*** of *Monohammus*? *Rhobetor* Newman, 1842, female: ‘Type [p, label circular, red framed]; Phil / Isla [p]; Monohammus? Rhobetor / fimbriatus Oliv. Pl 19 f 143 / Newman Entom. 276 [h]; Monohammus / rhobetor / Type Newm [h]; NHMUK014596273 [p]’.

#### Non-type material.

40 specimens. **Indonesia (33)**: Moluccas (1 male, 4 females); Amboina (2 males, 2 females); Ceram (5 males, 3 females); Celebes (1 female); Bouru [Borneo] (2 males, 1 female); Seram (4 females); Mausela (4 males, 4 females). **Philippines (4)**: Philippine Islands (1 male, 3 females). **India (1)**: Goram (1 female). **Country unknown (2)**: Bowring collection (1): maki. (1 male); Ent. Club (1 male).

### ﻿ Epepeotes desertus obscurus

Taxon classificationAnimaliaColeopteraCerambycidae

(Aurivillius, 1926)

6604300A-70E5-508B-AAE6-3E7C6AB2C278

Fig. 1g, h

Diochares desertus var. obscurus Aurivillius, 1926: 102. Type locality: Buru [Indonesia].
Epepeotes desertus s.- sp. obscurus : Breuning 1943: 229.
Epepeotes desertus ssp. obscurus : Breuning 1961: 326.
Epepeotes desertus obscurus : Barševskis 2020: 180.

#### Non-type material.

14 specimens. **Indonesia (14)**: Ceram (2 females); Mausela (5 males, 7 females).

#### Comments.

Breuning (1943) stated that the subspecies *E.desertusrhobetor* is distinguished from the subspecies *E.d.desertus* by the body usually smaller, the antennae more slender, the body markings yellow, and the elytral spots small and separate, with only a few individuals uniting into narrow transverse bands before and after the middle; the subspecies *E.d.obscurus* is separated from the nominate subspecies by the elytron with less concave apex and reduced body markings. However, the holotype of *E.d.rhobetor* (Fig. 1d) has the complete premedian transverse band and the postmedian band consisting of several large spots that are not fully fused, which is different from the description and illustration by Breuning (1943). Olivier (1808) illustrated the dorsal view of the species *Cerambixfimbriatus* Olivier, 1795 (Fig. 1c, now a synonym of the nominate subspecies), which differs from *E.d.rhobetor* and *E.d.obscurus* mainly in the large and complete premedian and postmedian transverse bands on the elytra. However, the body markings and size are variable in this species, which make it difficult to accurately distinguish the three subspecies. The taxonomic status of the three subspecies is expected to be resolved by further study of more type and non-type material, perhaps with the possible removal of the subspecies-level status.

### ﻿ Epepeotes luscus luscus

Taxon classificationAnimaliaColeopteraCerambycidae

(Fabricius, 1787)

B93DAB7C-43CF-57B5-90E9-D7B0217CF339

Fig. 2

Lamia lusca Fabricius, 1787: 139. Type locality: Siam [Thailand].
Cerambyx (Lamia) luscus : Gmelin 1790: 1836.
Epepeotes luscus : Pascoe 1866a: 249.
Epepeotes fumosus Pascoe, 1866b: 301. Type locality: Flores [Indonesia].
Epepeotes luscus var. enganensis Gahan, 1907: 80. Type locality: Engano [Indonesia].
Epepeotes luscus ab. flavomaculatus Aurivillius, 1924: 27. Type locality: Sumatra [Indonesia].
Epepeotes soembanicus Schwarzer, 1931: 66. Type locality: Mao Marroe: Ost-Soemba [Indonesia].
Epepeotes luscus m. *densemaculatus* Breuning, 1943: 223. Type locality: Birmanie [Burma].
Epepeotes luscus m. *ochreosticticus* Breuning, 1943: 223. Type locality: Nicobar Island [India].
Epepeotes luscus m. *interruptus* Breuning, 1943: 223. Type locality: Nias Island [Indonesia].
Epepeotes luscus alorensis Breuning, 1960: 29. Type locality: Alor Island [Indonesia].
Epepeotes luscus var. subuniformis Gilmour & Breuning, 1963: 125. Type locality: Sumatra: P. Sebuku [Indonesia].

#### Type material.

**Thailand. *Holotype***, male: ‘Type [p, label circular, red framed]; Lam. Lusca / Fabr. Mant. Ins. n. 35 [h]; NHMUK014596790 [p]’. **Indonesia. *Holotype*** of *Epepeotesfumosus* Pascoe, 1866, female: ‘Type [p, label circular, red framed]; Flores [h, label oval, dark green]; Pascoe / Coll. / 93–60 [p]; Epepeotes / fumosus / Type Pasc [h]; Epepeotes / fumosus / Flores Pa [h]; NHMUK014596286 [p]’; **Indonesia.** One ***syntype*** of Epepeotesluscusvar.enganensis Gahan, 1907, male: ‘Sumatra / Engano Is. [p]; Doherty [p]; 67584 [h]; Fry Coll. / 1905. 100. [p]; Epepeotes / luscus. F. / v. enganesis / ♂ Type Gahan [h], NHMUK014596282’; **Indonesia. *Syntypes*** of Epepeotesluscusvar.enganensis Gahan, 1907, two females: ‘Sumatra / Engano Is. [p]; Doherty [p]; 67584 [h]; Fry Coll. / 1905. 100. [p]’.

#### Non-type material.

264 specimens. **Indonesia (77)**: Gunung Perak (1 female); Java (15 males, 7 females); Sumatra (27 males, 16 females); Borneo (4 males, 3 females); Tambang Sawah (1 male, 1 female); Lom. [Lombok] (1 male); Nias (1 female); **Malaysia (135)**: N. Borneo (4 males, 3 females); Dindings (1 female); Malacca (1 male); Penang (2 males, 5 females); Perak (6 males, 4 females); Pahang (2 females); Malay (2 males, 1 female); Malay Peninsula (15 males, 12 females); Malaya (5 males, 5 females); Kinabalu (1 male), Kumching (6 males, 6 females); Malay States (4 males, 1 female); Sarawak (26 males, 16 females); Bukit kutu (1 male, 1 female); Gunung Angsi (1 female); Tenasserim Hill (2 males); Siam: Malay states (1 female); Pulau Tioman (1 male). **Cambodia (2)**: Camboja (2 females). **India (7)**: India (1 female), India Orient (3 males, 1 female); Jalor (2 males). **Myanmar (5)**: Burmah (2 females), Rangoon (3 males). **Thailand (6)**: Peninsular Siam, Nakon Sri Tammarat (2 males, 1 female), Siam, Me Song Forest (1 female); Khao Ram, Siam (1 female); Ronpilum (1 female). **Laos (2)**: Phou Tiou (2 males). **Singapore (10)**: Singapore (3 male, 5 females), Singapore Island (2 females). **Country unknown (20)**: Bowring–Chevrolat collection (2): locality label not present (1 male); locality label unreadable (1 male). Ex. F. M. S. Museum (4): Gap (1 male); Kia moo (1 female); locality label unreadable (1 female); locality label not present (1 male). Pascoe collection (1): locality label unreadable (1 male). G. Bryant Collecton (3): Maling (3 males). E. I. C. (3): locality label not present (2 males, 1 female). Ind. Mus. (1): locality label not present (1 female). Imp. Bur. Ent. (1): Th Gap (1 male). Timol (1 male). Thassauaddy (1 male). Kne (1 female). locality label unreadable (1 male). locality label not present (1 male).

#### Comments.

**Figure 2.**
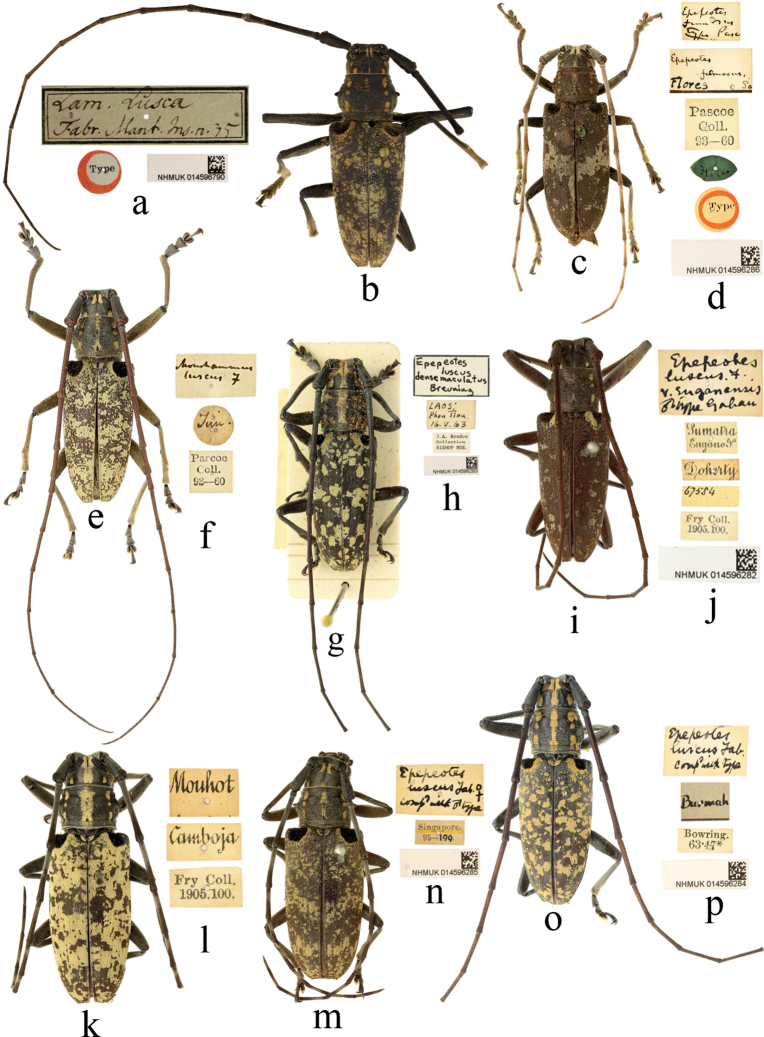
Habitus of *Epepeotesluscusluscus* (Fabricius, 1787) **a, b** holotype of *Lamialusca* Fabricius, 1787 **c, d** holotype of *Epepeotesfumosus* Pascoe, 1866 **i, j** syntype of Epepeotesluscusvar.enganensis Gahan, 1907 **b, e, g, i, o** male **c, k, m** female **f, h, l, n, p** labels.

Two specimens from Camboja [Cambodia] (Fig. 2k, l) and 10 specimens from Singapore (Fig. 2m, n) were found in the collection, which represent the new country record respectively.

### ﻿ Epepeotes lateralis

Taxon classificationAnimaliaColeopteraCerambycidae

(Guérin-Ménevilie, 1831)

E1580CED-D06C-545F-85CA-C6362D1794D6

Fig. 3a–e

Monochamus lateralis Guérin-Ménevilie, 1831: 113. Type locality: unknown.
Leprodera spinosa Thomson, 1857: 179. Type locality: Java [Indonesia].
Epepeotes meridianus Pascoe, 1866: 302. Type locality: Java, Sumatra, Tondano [Indonesia]; Sarawak [Malaysia], Singapore.
Epepeotes lateralis : Aurivillius 1922: 79.
Epepeotes lateralis var. niasica Aurivillius, 1923: 452. Type locality: Nias [Indonesia].
Epepeotes spinosus m. *bimaculatus* Breuning, 1943: 225. Type locality: Java [Indonesia].

#### Type material.

**Indonesia. *Syntype*** of *Epepeotesmeridianus* Pascoe, 1866, male: ‘Type [p, label circular, red framed]; Java [h, label oval, dark green]; Epepeotes / meridianus / type Pasc. [h]; Epepeotes / meridianus / Java P [h]; NHMUK014596276 [p]’.

#### Non-type material.

72 specimens. **Indonesia (31)**: Nias (2 males, 2 females); Java (7 males, 10 females); Sumatra (3 males, 3 females); Borneo (1 male, 3 females). **Malaysia (25)**: Sarawak (12 males, 8 females); Penang (1 male); Perak (2 females); Siamess Malay states (1 male); Malay states (1 female). **Singapore (3)**: Singapore (2 males, 1 female). **Country unknown (13)**: G. Bryant collection (6): Maling (4 males, 2 females), Bowring–Chevrolat collection (4): locality label not present (2 males, 1 female); locality unreadable (1 female). E. I. C. (1): locality label not present (1 male). locality label not present (2 females).

#### Comments.

**Figure 3.**
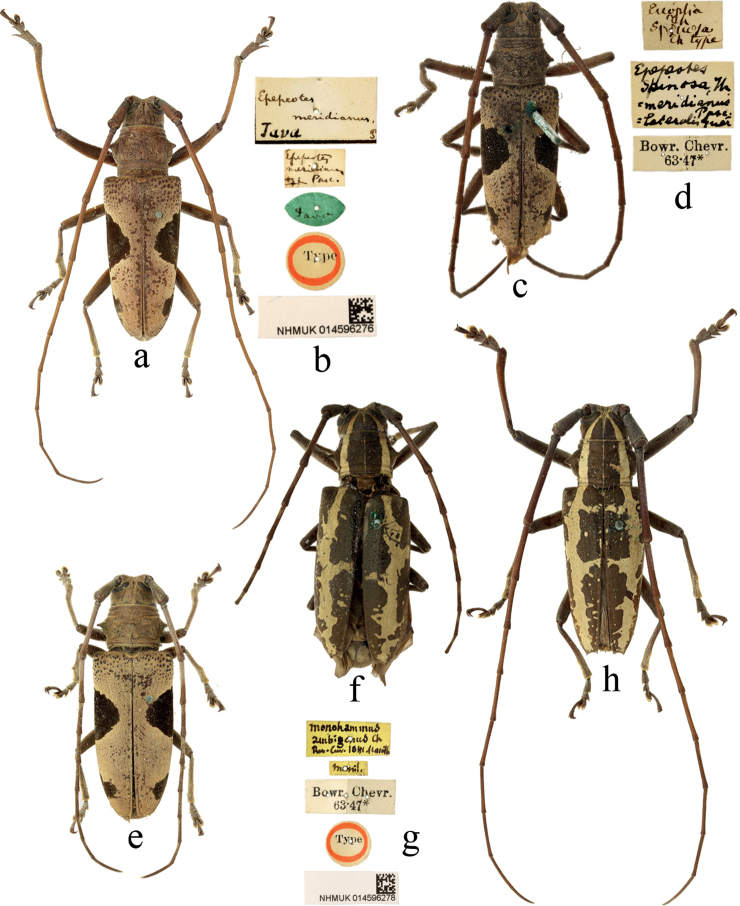
Habitus of *Epepeotes* spp. **a–e***Epepeoteslateralis* (Guérin-Méneville, 1831) **f–h***Epepeotesambigenusambigenus* (Chevrolat, 1841) **a, b** syntype of *Epepeotesmeridianus* Pascoe, 1866 **f, g** holotype **a, c, h** male **e, f** female.

There is a specimen from Bowring–Chevrolate collection labeled with a handwritten “Type” label [Fig. 3c, d]: ‘Euoplia / Th / spinosa / Th type’. ‘Th’ should be the abbreviation of ‘Thomson’. However, the genus *Euoplia* was erected by Hope (1839), and Thomson (1857) published this species with the name of ‘*Leproderaspinosa*’, and the type specimens are deposited in the Muséum national d’Histoire naturelle, Paris. Obviously, this is a labelling error by a curator and it is not the type specimen.

### ﻿ Epepeotes ambigenus ambigenus

Taxon classificationAnimaliaColeopteraCerambycidae

(Chevrolat, 1841)

D6028D39-BE61-5A8E-A395-AFC7736FF13F

Fig. 3f–h

Monohammus ambigenus Chevrolat, 1841: 228. Type locality: Manila [Philippines].
Diochares ambigenus : Pascoe 1866: 303.
Mengelotes ambiguus : Dillon and Dillon 1941: 43. Type locality: Mexico.
Epepeotes ambigenus : Breuning 1943: 228.
Epepeotes ambigenus ambigenus : Hua 2002: 207.

#### Type material.

**Philippines. *Holotype***, female: ‘Type [p, label circular, red framed]; Manil. [h]; Monohammus / ambigenus Ch / Rev. Zoo, 1841 Manille [h] / Bowr. Chevr. /, 63· 47* [p] / NHMUK014596278 [p]’.

#### Non-type material.

22 specimens. **Philippines (18)**: Philippine Islands (8 males 4 females), Philippines (1 male, 1 female), PHIL. (1 male, 1 female), Luzon (1 male); Albay (1 female); **Indonesia (1)**: Java (1 male); **Country unknown (3)**: Corla (1 male); locality label not present (1 male, 1 female).

### ﻿ Epepeotes plorator plorator

Taxon classificationAnimaliaColeopteraCerambycidae

(Newman, 1842)

B9CCD20A-5CC1-546C-AFAE-904005C70C35

Fig. 4a, b, e

Monohammus plorator Newman, 1842: 276. Type locality: Manille [Philippines].
Rhamses vitticollis Thomson, 1878: xviii. Type locality: Borneo [Indonesia].
Pelargoderus vitticollis : Ritsema 1885: 44.
Diochares mindanaonis Heller, 1915: 240. Type locality: Mindanao, Davao [Philippines].
Epepeotes plorator : Heller 1916: 306.
Epepeotes multinotatus Pic, 1925: 19. Type locality: Japan.
Epepeotes plorator m. mindanaonis: Breuning 1943: 225.
Epepeotes spinosoides Breuning, 1980: 170. Type locality: Mindanao [Philippines].

#### Type material.

**Philippines. *Holotype***, male: ‘Type [p, label circular, red framed]; Phil / Isla [p]; Monohammusplorator / Newman Entomol 276 [h]; Monohammus / plorator / Type Newm. [h]; NHMUK014596277’.

**Figure 4.**
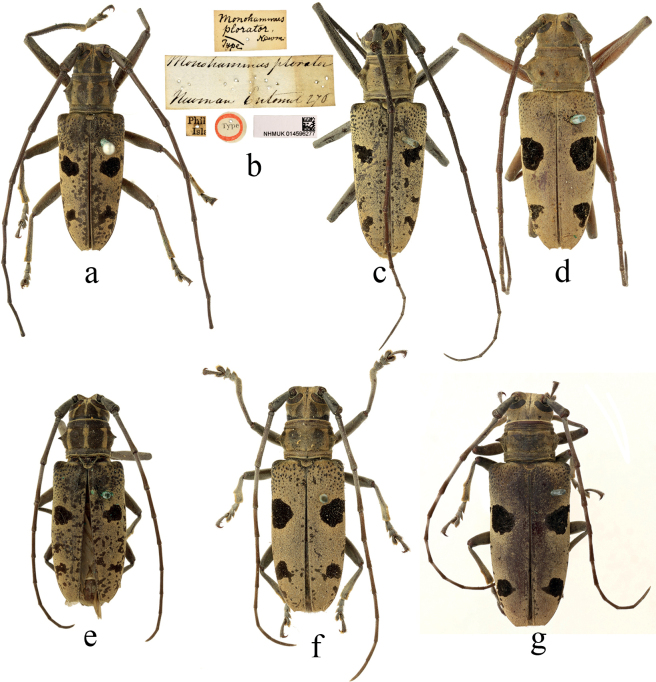
Habitus of *Epepeotesplorator* (Newman, 1842) **a, b, e***Epepeotesploratorplorator* (Newman, 1842) **c, f***Epepeotesploratorsanghiricus* Breuning, 1938 **d, g***Epepeotesploratorcelebensis* Aurivillius, 1922 **a, b** holotype **a,c, d** male **e, f, g** female.

#### Non-type material.

3 specimens. **Philippines (3)**: Philippine Islands (1 male, 2 females).

### ﻿ Epepeotes plorator celebensis

Taxon classificationAnimaliaColeopteraCerambycidae

Aurivillius, 1922

9A4D476B-117E-5F3C-BA6A-68394ABC135F

Fig. 4d, g

Epepeotes plorator var. celebensis Aurivillius, 1922: 80. Type locality: Celebes [Indonesia].
Epepeotes plorator s.- sp. celebensis : Breuning 1943: 225.
Epepeotes plorator ssp. celebensis : Breuning 1961: 326.
Epepeotes plorator celebensis : Fahri and Sataral 2015: 152.

#### Non-type material.

14 specimens. **Indonesia (10)**: Celebes (5 males, 2 females), Borneo (1 male), New Guinea (1 female), Macassar (1 male); **Country unknown (4)**: Bowring collection (1), locality label not present or unclear (1 male). Pascoe collection (3): Tond. (2 males, 1 female).

### ﻿ Epepeotes plorator sanghiricus

Taxon classificationAnimaliaColeopteraCerambycidae

Breuning, 1938

5F0E6849-BEDC-586C-8D66-5FE0713AD758

Fig. 4c, f

Epepeotes sanghiricus Breuning, 1938: 183. Type locality: Sanghir [Indonesia].
Epepeotes plorator ssp. sanghiricus Breuning, 1970: 376. Type locality: Sanghir [Indonesia].

#### Non-type material.

18 specimens. **Indonesia (12)**: Celebes (7 males, 2 female), Borneo (1 male), Macassar (1 female), Koepang (1 female). **Country unknown (6)**: Pascoe collection (6): Macanor (2 males), Mak. (1 female, 3 females).

#### Comments.

Subspecies *E.p.plorator* (Newman, 1842) is different from subspecies *E.p.celebensis* Aurivillius, 1922 and *E.p.sanghiricus* Breuning, 1938 by the pronotum and vertex mostly black, with three distinct yellow longitudinal stripes, *E.p.celebensis* Aurivillius, 1922 is distinguished from *E.p.sanghiricus* Breuning, 1938 by the pronotum and vertex completely covered with pubescence, the elytra seldom with small spots among the four main black spots, while in *E.p.sanghiricus* Breuning, the pronotum and vertex is decorated with two inconspicuous black longitudinal strips, sometimes reduced to black spots, the elytra usually is dotted with some small and irregular black spots in addition to the main markings.

This subspecies was described twice by Breuning (1938, 1970), the first time as species *Epepeotessanghiricus* (type in Naturhistorisches Museum Basel, Switzerland) and the second time as subspecies Epepeotesploratorssp.sanghiricus (type in Muséum national d’Histoire naturelle, Paris, France).

### ﻿ Epepeotes commixtus

Taxon classificationAnimaliaColeopteraCerambycidae

(Pascoe, 1859)

C43A786A-9EB5-5F3C-B1D1-2224BFEA9CC5

Fig. 5a, b

Monohammus commixtus Pascoe, 1859: 42. Type locality: Ceylon [Sri Lanka].
Epepeotes commixtus : Breuning 1943: 227.

#### Type material.

**Sri Lanka. *Holotype***, male: ‘Type [p, label circular, red framed]; Ceylon [h, label circular]; Monohammus / commixtus / Pascoe. [h]; NHMUK014596281 [p]’.

#### Non-type material.

8 specimens. **Sri Lanka (6)**: Ceylon (1 male, without head, 5 females). **Malaysia (1)**: Sarawak (1 female). **Country unknown (1)**: Bowring–Chevrolat collection (1): locality label not present (1 female).

**Figure 5.**
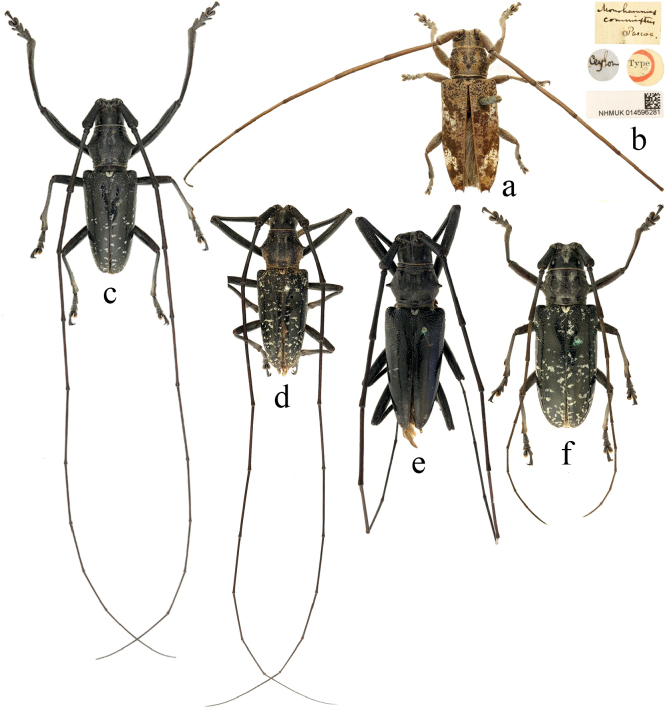
Habitus of *Epepeotes* spp. **a, b***Epepeotescommixtus* (Pascoe, 1859) **c–f***Epepeotesceramensis* (Thomson, 1861) **a, b** holotype **a, c–e** male **f** female.

### ﻿ Epepeotes ceramensis

Taxon classificationAnimaliaColeopteraCerambycidae

(Thomson, 1861)

A6FA6685-AF46-5BC6-9315-A2457B8AEF98

Fig. 5c–f

Rhamses ceramensis Thomson, 1861: 361. Type locality: Ceram [Indonesia].
Pelargoderus Ceramensis[sic]: Pascoe 1866: 279.
Epepeotes ceramensis : Breuning 1943: 148, 226.

#### Non-type material.

14 specimens. **Indonesia (14)**: Ceram (7 males, 7 females).

#### Comments.

The light-colored spots on the elytra are variable, from very distinct to nearly invisible (Fig. 5d, e).

### ﻿ Epepeotes diversus

Taxon classificationAnimaliaColeopteraCerambycidae

Pascoe, 1866

82D2D3D1-9856-57B3-BB19-6D6C882F5DB5

Fig. 6a–c

Epepeotes diversus Pascoe, 1866: 302. Type locality: Kei [Indonesia].

#### Type material.

**Indonesia. *Holotype***, male: ‘Type [p, label circular, red framed]; Ké / 3. [h, label circular]; Pascoe / Coll. / 93–60 [p]; diversus [h]; Epepeotes / diversus / Ké Is. Pas [h]; NHMUK014596279 [p]’.

**Figure 6.**
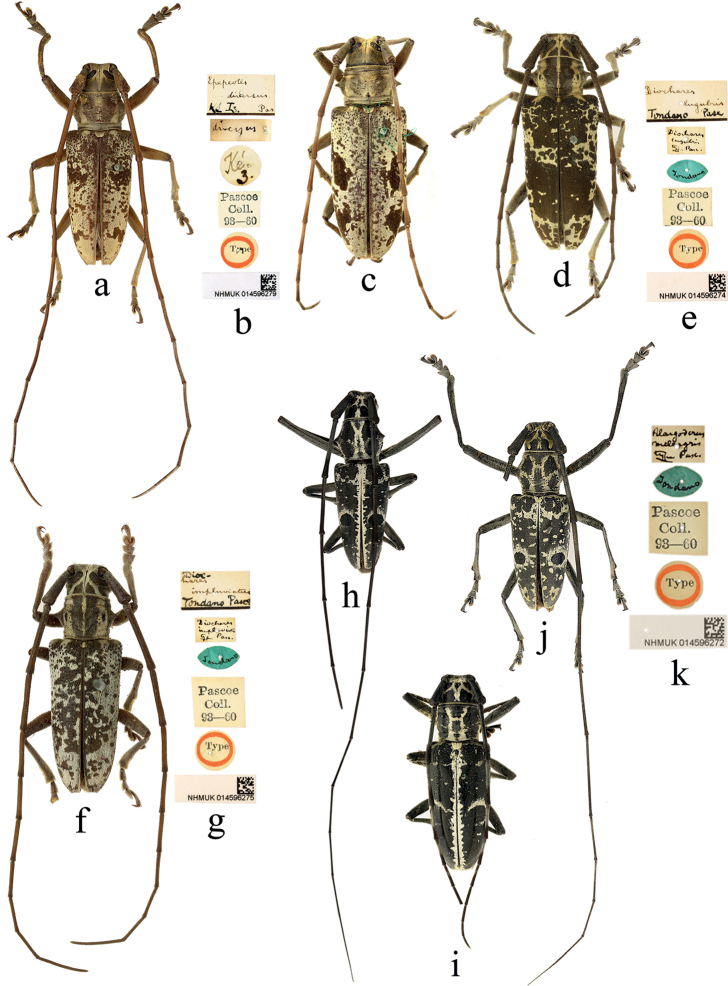
Habitus of *Epepeotes* spp. **a–c***Epepeotesdiversus* Pascoe, 1866 **d–g***Epepeoteslugubris* (Pascoe, 1866) **h–k***Epepeotesmeleagris* (Pascoe, 1866) **f, g** holotype of *Diocharesimpluviatus* Pascoe, 1866 **a, b, d, e, j, k** holotype **a, f, h, j** male **c, d, i** female.

#### Non-type material.

1 specimen. **Indonesia (1)**: Key [Kei] (1 female).

### ﻿ Epepeotes lugubris

Taxon classificationAnimaliaColeopteraCerambycidae

(Pascoe, 1866)

A5615ECE-C614-56E7-A303-EFABB4164E16

Fig. 6d–g

Diochares lugubris Pascoe, 1866: 304. Type locality: Tondano [Indonesia].
Diochares impluviatus Pascoe, 1866: 305. Type locality: Tondano [Indonesia].
Epepeotes lugubris : Breuning 1943: 230.

#### Type material.

**Indonesia. *Holotype***, female: ‘Type [p, label circular, red framed]; Tondano [h, label oval, dark green]; Pascoe / Coll. / 93–60 [p]; Diochares / lugubris / Type Pasc. [h] / Diochares / lugubris / Tondano Pasc [h]; NHMUK014596274 [p]’; **Indonesia. *Holotype*** of *Diocharesimpluviatus* Pascoe, 1866, male: ‘Type [p, label circular, red framed]; Tondano [h, label oval, dark green]; Pascoe / Coll. / 93–60 [p]; Diochares / impluviatus / Type Pasc. [h] / Dioc- / hares / impluviatus / Tondano Pasc [h]; NHMUK014596275’.

#### Non-type material.

1 specimen. **Indonesia (1)**: Celebes (1 female).

### ﻿ Epepeotes meleagris

Taxon classificationAnimaliaColeopteraCerambycidae

(Pascoe, 1866)

A0D24B1B-D0E4-5E01-8E1A-1D36625BC41E

Fig. 6h–k

Pelargoderus meleagris Pascoe, 1866: 279. Type locality: Tondano [Indonesia].
Epepeotes meleagris : Breuning 1943: 226.

#### Type material.

**Indonesia. *Holotype***, male: ‘Type [p, label circular, red framed]; Tondano [h, label oval, dark green]; Pascoe / Coll. / 93–60 [p]; *Pelargoderus* / *meleagris* / Type Pasc. [h]; NHMUK014596272’.

#### Non-type material.

10 specimens. **Indonesia (8)**: Celebes (6 males, 2 females). **Country unknown (2)**: ex H. J. Carter (2 males).

#### Comments.

Dorsal markings of this species are reduced in some male individuals, as in females, with the eyelike black spot on each elytron is without conspicuous posterior pale margins and spots along the suture are uniting into a white line.

### ﻿ Epepeotes vestigialis vestigialis

Taxon classificationAnimaliaColeopteraCerambycidae

Pascoe, 1866

45B3E5DD-A88F-5BD6-AA1A-D94A3E767559

Fig. 7a–c

Epepeotes vestigialis Pascoe, 1866: 301. Type locality: Sarawak [Malaysia].

#### Type material.

**Malaysia. *Holotype***, male: ‘Type [p, label circular, red framed]; Sarawak [h, label oval, dark green]; Epepeotes / vestigialis / Type Pasc. [h]; Epepeotes / vestigialis. / Sarawak Pas [h]; NHMUK014596265 [p]’.

**Figure 7.**
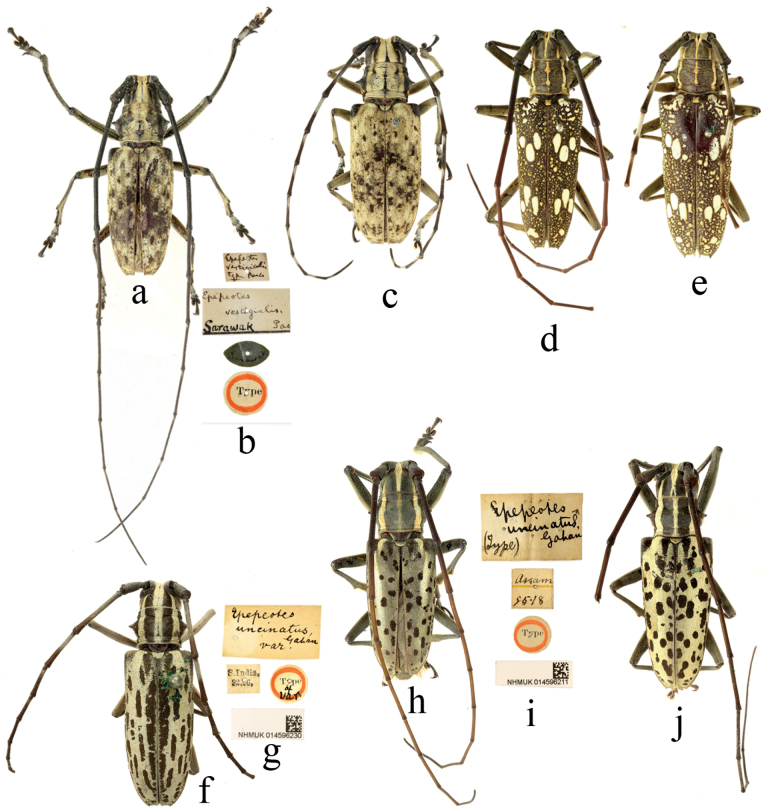
Habitus of *Epepeotes* spp. **a–c***Epepeotesvestigialisvestigialis* Pascoe, 1866 **d, e***Epepeotesschlegelii* Lansberge, 1884 **f, g***Epepeotesuncinatuslineatopunctatus* Breuning, 1960 stat. Resurrected **h, i***Epepeotesuncinatusuncinatus* Gahan, 1888, lectotype **j***Epepeotesuncinatusuncinatus* m. *salvazai* Pic, 1925 **a, d, h, j** male **c, e, f** female.

#### Non-type material.

9 specimens. **Indonesia (2)**: Borneo (2 males). **Malaysia (5)**: Sarawak (1 female), Perak (2 males, 1 female), Malay Penin. (1 female). **Laos (1)**: Parkading (1 male). **Country unknown (1)**: Ex. F. M. S. Museum. B.M. 1955–354, locality label unreadable (1 female).

### ﻿ Epepeotes schlegelii

Taxon classificationAnimaliaColeopteraCerambycidae

Lansberge, 1884

71AC7FFF-587F-5ACC-A0E8-F4CCBBD56781

Fig. 7d, e

Epepeotes Schlegelii [sic] Lansberge, 1884: 90. Type locality: Sumatra [Indonesia].
Epepeotes diversemaculatus Schwarzer, 1927: 60. Type locality: Gunung Singalang [Indonesia].
Epepeotes schlegelii : de Jong 1942: 28 (synonymy).

#### Non-type material.

5 specimens. **Indonesia (5)**: Sumatra (4 males), Sungei Kumbang (1 female)

### ﻿ Epepeotes uncinatus uncinatus

Taxon classificationAnimaliaColeopteraCerambycidae

Gahan, 1888

345994E5-B7FE-5836-AEDB-2B0524DD6F64

Fig. 7h–j

Epepeotes uncinatus Gahan, 1888: 271. Type locality: Assam [India].
Epepeotes uncinatus v. *salvazai* Pic, 1925: 18. Type locality: Laos.
Pseudopsacothea lineata Pic, 1944: 14. Type locality: Annam [Vietnam].

#### Type material.

**India. *Lectotype***, male (designated herein): ‘Type [p, label circular, red framed]; Assam / 95·18[h]; Epepeotes / ♂ / uncinatus / (Type) Gahan [h]; NHMUK014596211’.

#### Non-type material.

47 specimens. **India (28)**: India (1 female); India, Bowring collection (1 male 1 female); India Orient (1 male 1 female); N. India, Bowring collection (1 male); S. India kanara (1 female); Assam (3 males, 7 females); Cachar [Assam] (1 male); Cherra Poonjee [now Cherrapunji], E. I. C. (1 male); Darjeeling (1 male, 1 female); Gopaldhara (2 females); Lebong (1 female); Shilong (1 male, 2 females); Sikkim (1 female). **Burma (6)**: Burma, Fry collection (1 male); Chin Hills (1 male, 1 female); Upper Burma, Nam Tamai Valley, 25. viii. 1938 (2 males, 1 female). **Bangladesh (7)**: Silhet [now Sylhet], Bowring/Chevrolat collection (1 male); Silhet, Bowring collection (1 male, 1 female); Sehet [now Sylhet] (1 male, 1 female); Silhet, E. I. C (1 male); Silhet, Pascoe collection (1 female). **Country unknown (6)**: Bowring–Chevrolat collection (1), locality label not present (1 female); Dharg, Bowring collection (1 male, 1 female); Silcurl (1 male, 1 female); locality label not present (1 female).

#### Comments.

One syntype was confirmed in the collection. Herein we designated it as the lectotype, which has been labeled with a red-framed circular printed label and a handwritten identification label, both marked with ‘Type’. This species is recorded in Bangladesh for the first time.

### ﻿ Epepeotes uncinatus lineatopunctatus

Taxon classificationAnimaliaColeopteraCerambycidae

Breuning, 1960, stat. resurrected

C99A17ED-23F1-5750-9AD7-4192C57964F0

Fig. 7f, g

Epepeotes uncinatus lineatopunctatus Breuning, 1960: 29. Type locality: Travancore [India].
Epepeotes uncinatus m. lineatopunctatus Breuning, 1961: 325.

#### Non-type material.

12 specimens. **India (12)**: Nilgiri Hills (4 males); S. India (1 male, 6 females); Upper Assam (1 male).

#### Comments.

A female specimen from South India (Fig. 7f, g) was mislabeled with a red-framed circular printed type label in the collection, which represents actually a subspecies of *E.uncinatus* Gahan, 1888, not the type specimen. Breuning (1960) described the subspecies *Epepeotesuncinatuslineatopunctatus*, then he (1961) regarded it as a morph of *E.uncinatus* Gahan. However, this subspecies is distinctly different from the nominate subspecies by the elytron mostly with conspicuous, short, longitudinal black spots, rather than rounded to oblong spots, which are almost united into a long longitudinal strip along the suture, the vertex and pronotum with more expanded median longitudinal strip. On this basis, herein we restore it to subspecies-level status.

### ﻿ Epepeotes andamanicus

Taxon classificationAnimaliaColeopteraCerambycidae

Gahan, 1893

6CCB60DF-70F5-583B-BE92-732CFBE601F4

Fig. 8a–c

Epepeotes andamanicus Gahan, 1893: 380. Type locality: Andaman Islands [India].

#### Type material.

**India. *Lectotype***, female (designated herein): ‘Type [p, label circular, red framed]; Andaman. Is. / 61–61. [p]; Epepeotes / andamanicus / Type ♀ Gahan [h]; NHMUK014394970 [p]’.

#### Non-type material.

8 specimens. **India (8)**: Andaman Islands (2 males, 6 females).

#### Comments.

**Figure 8.**
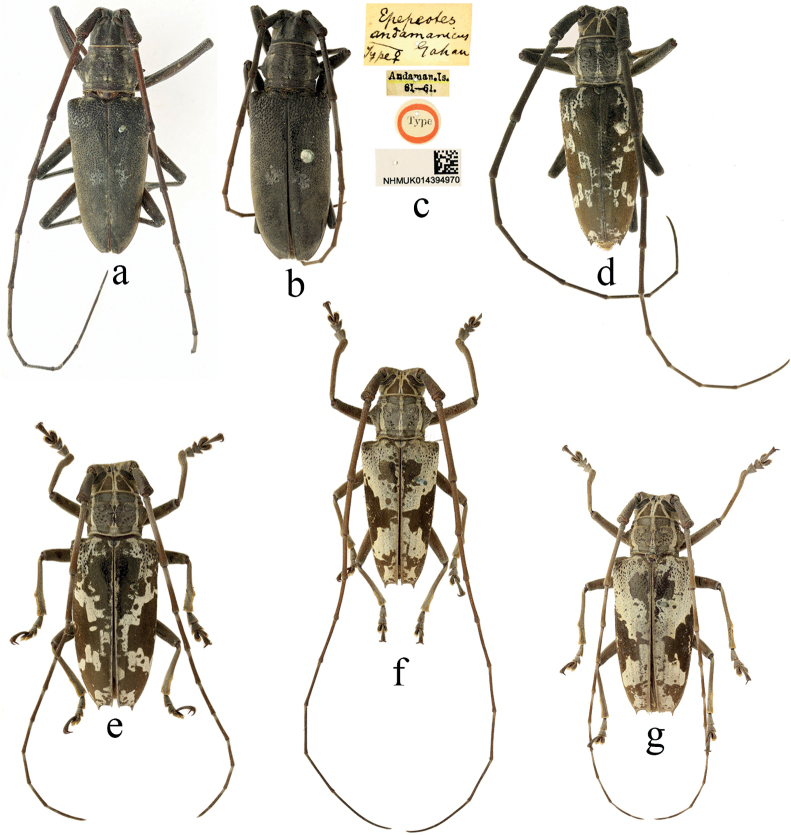
Habitus of *Epepeotes* spp. **a–c***Epepeotesandamanicus* Gahan, 1893 **d–g***Epepeotestaeniotinustaeniotinus* (Heller, 1924) **b, c** lectotype **a, d, f** male **b, e, g** female.

One syntype was confirmed in the collection. Herein we designated it as the lectotype (Fig. 8b, c), which has been labeled with a red-framed circular printed label and a handwritten identification label, both marked with ‘Type’.

### ﻿ Epepeotes taeniotinus taeniotinus

Taxon classificationAnimaliaColeopteraCerambycidae

(Heller, 1924)

4ADAF509-EBB1-5D05-93CD-EEDB8FEE4AED

Fig. 8d–g

Diochares desertus taeniotinus Heller, 1924: 424. Type locality: Samar [Philippines].
Epepeotes taeniotinus : Breuning 1943: 229.
Epepeotes taeniotinus taeniotinus : Barševskis 2020: 181.

#### Non-type material.

27 specimens. **Indonesia (18)**: Maluccas [Moluccas] (2 males, 3 females); Celebes (1 male); Batchian (1 male, 3 females); Ternate (3 males, 3 females); Morty (2 females). **Country unknown (9)**: H.L. Andrewes Bequest. (4): locality label unclear (3 males, 1 female); Pascoe collection (5): Mai (1 female); Gil (1 female); Maki (1 male, 1 female); locality label unreadable (1 female).

#### Comments.

The pale markings on elytra are variable, ranging from occupying most of the elytra to being almost a narrow X-like stripe.

### ﻿ Epepeotes strandi

Taxon classificationAnimaliaColeopteraCerambycidae

(Breuning, 1935)

F5D5E617-74A7-50BE-8553-56EC44D9F0CF

Fig. 9a–f

Pelargoderus strandi Breuning, 1935: 253. Type locality: Andaman [India].
Epepeotes quadriplagiatus Breuning, 1936: 296. Type locality: Andaman [India].
Epepeotes strandi : Breuning 1943: 148, 226 (synonymy).

#### Type material.

**India. *Syntypes*** of *Epepeotesquadriplagiatus* Breuning, 1936: one male: ‘Type [p, label circular, red framed]; Andaman Is. [p]; And. Is. [p]; Epepeotes / 4 plagiatus / mihi Typ. [h] / det. Breuning [p]; NHMUK014394973 [p]’; one male: ‘Andaman Is. [p]; And. Is. [p]; Epepeotes / quadriplagiatus / mihi Typ! [h] / det. Breuning [p]; NHMUK014394960 [p]’. **India. *Paratypes*** of *Epepeotesquadriplagiatus* Breuning, 1936: one female: ‘Andaman / Islands. [p]; Atkinson / Coll. / 92–3 [p]; Epepeotes / 4 plagiatus / Breun. Paratyp [h]; NHMUK014394962 [p]’; One male: ‘91–17 [p]; Epepeotes / 4 plagiatus / Breun. Paratyp [h]; NHMUK014394963 [p]’.

#### Comments.

**Figure 9.**
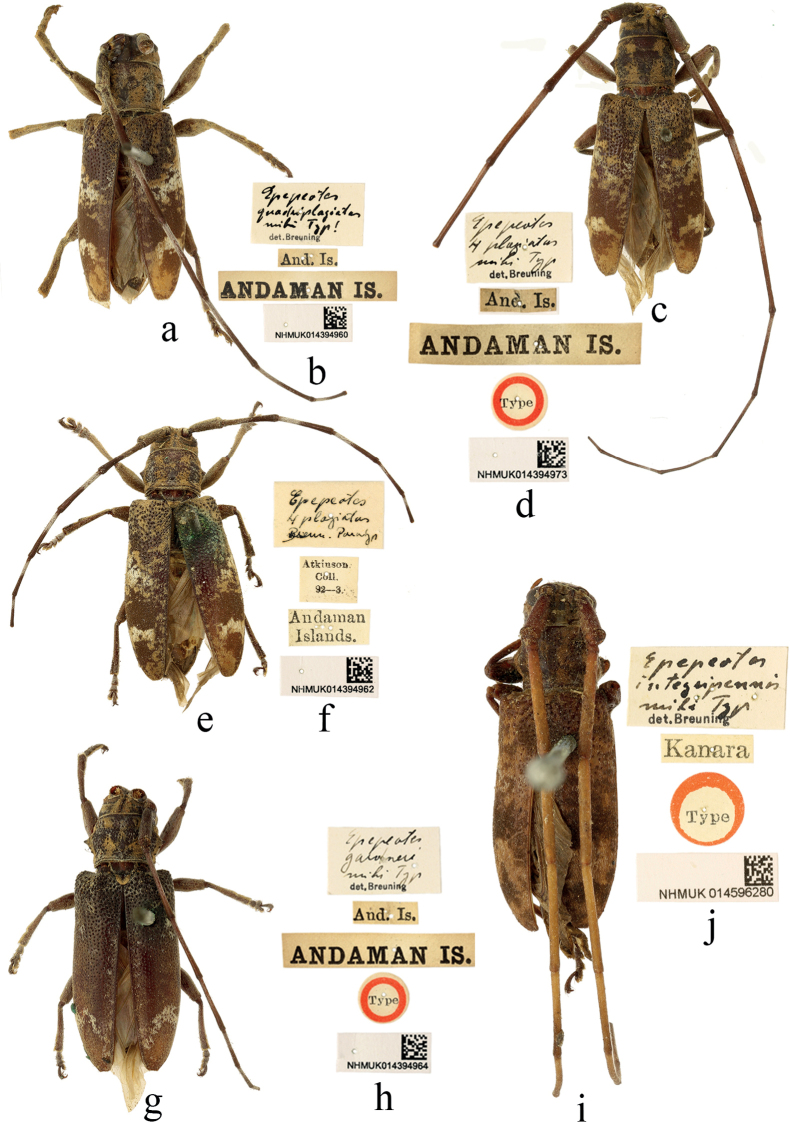
Habitus of *Epepeotes* spp. **a–f** types of *Epepeotesquadriplagiatus* Breuning, 1936 **g, h** holotype of *Epepeotesgardneri* Breuning, 1936 **i, j** holotype of *Epepeotesintegripennis* Breuning, 1940 **a, c, i** male **e, g** female.

A total of four type specimens of *E.quadriplagiatus* are found in the collection, of which two male specimens are marked with ‘Type’ and the others (one male and one female) are marked with ‘Paratype’ by Breuning’s handwriting (Fig. 9b, d, f). Though Breuning (1936) originally designated one male as the type, the two male specimens labeled as ‘type’ by Breuning become de facto syntypes.

### ﻿ Epepeotes gardneri

Taxon classificationAnimaliaColeopteraCerambycidae

Breuning, 1936

2F0F4800-B9A0-5D61-B203-B077F95E4E8D

Fig. 9g, h

Epepeotes gardneri Breuning, 1936: 287. Type locality: Andaman [India].

#### Type material.

**India. *Holotype***, female: ‘Type [p, label circular, red framed]; Andaman Is. [p]; And. Is. [p]; Epepeotes / gardneri / mihi Typ. [h] / det. Breuning [p]; NHMUK014394964 [p]’.

### ﻿ Epepeotes integripennis

Taxon classificationAnimaliaColeopteraCerambycidae

Breuning, 1940

35F99D79-BA34-5837-9727-0ABFF24ABDD0

Fig. 9i, j

Epepeotes integripennis Breuning, 1940: 123. Type locality: Kanara [India].

#### Type material.

**India. *Holotype***, male: ‘Type [p, label circular, red framed]; Kanara [p]; Epepeotes / integripennis / mihi Typ [p] / det. Breuning [p]; NHMUK014596280 [p]’.

## Supplementary Material

XML Treatment for Epepeotes desertus desertus

XML Treatment for Epepeotes desertus rhobetor

XML Treatment for Epepeotes desertus obscurus

XML Treatment for Epepeotes luscus luscus

XML Treatment for Epepeotes lateralis

XML Treatment for Epepeotes ambigenus ambigenus

XML Treatment for Epepeotes plorator plorator

XML Treatment for Epepeotes plorator celebensis

XML Treatment for Epepeotes plorator sanghiricus

XML Treatment for Epepeotes commixtus

XML Treatment for Epepeotes ceramensis

XML Treatment for Epepeotes diversus

XML Treatment for Epepeotes lugubris

XML Treatment for Epepeotes meleagris

XML Treatment for Epepeotes vestigialis vestigialis

XML Treatment for Epepeotes schlegelii

XML Treatment for Epepeotes uncinatus uncinatus

XML Treatment for Epepeotes uncinatus lineatopunctatus

XML Treatment for Epepeotes andamanicus

XML Treatment for Epepeotes taeniotinus taeniotinus

XML Treatment for Epepeotes strandi

XML Treatment for Epepeotes gardneri

XML Treatment for Epepeotes integripennis

## ﻿Additional information

### Author ORCIDs

Guanglin Xie https://orcid.org/0000-0003-3273-8985

Maxwell V. L. Barclay https://orcid.org/0000-0003-4989-2014

Wenkai Wang https://orcid.org/0000-0002-4482-076X

### Data availability

All of the data that support the findings of this study are available in the main text.
